# Beyond the Spontaneous Breathing Trial: Echocardiographic and Integrated Ultrasound Assessment During Weaning from Mechanical Ventilation

**DOI:** 10.3390/diagnostics16111709

**Published:** 2026-06-02

**Authors:** Saeed Torabi, Philipp K. Omuro

**Affiliations:** Department of Anaesthesiology and Intensive Care Medicine, University Hospital of Cologne, Faculty of Medicine, University of Cologne, 50937 Cologne, Germany; philipp.omuro@uk-koeln.de

**Keywords:** mechanical ventilation, weaning, echocardiography, diastolic dysfunction, left atrial pressure, right ventricular dysfunction, lung ultrasound, diaphragm ultrasound, intensive care unit

## Abstract

Background/Objectives: Weaning failure from mechanical ventilation affects 10–20% of critically ill patients. Cardiovascular dysfunction—particularly diastolic dysfunction with elevated left atrial pressure (LAP)—underlies up to 50–60% of failed spontaneous breathing trials (SBTs) and frequently remains undetected without targeted echocardiographic assessment. This narrative review synthesises current evidence on the echocardiographic evaluation of weaning failure, with emphasis on LAP estimation, right ventricular (RV) dysfunction, and the integration of lung and diaphragm ultrasound. Methods: A structured literature search of PubMed/MEDLINE and EMBASE was performed for publications from January 2000 to April 2026, supplemented by hand-searching of reference lists and current society guidelines. This article is reported as a narrative review; no formal systematic review protocol was registered. A qualitative synthesis emphasising pathophysiological mechanisms, echocardiographic phenotypes, and clinical applicability was performed. Results: Positive pressure ventilation with PEEP provides active LV afterload reduction; extubation abruptly removes this unloading and may precipitate acute filling pressure elevation in vulnerable patients. Multiparametric echocardiographic LAP assessment—integrating the E/e’ ratio, deceleration time, and pulmonary vein flow—supports pre-extubation risk stratification. The dynamic PEEP reduction test, although not yet standardised or multicentre-validated, may identify patients with load-dependent cardiac decompensation before extubation. RV dysfunction is present in 20–50% of ventilated patients and worsens weaning outcomes through ventricular interdependence. Complementary lung ultrasound B-line quantification and diaphragm thickening fraction assessment together support a phenotype-specific diagnostic approach. Conclusions: A structured multimodal ultrasound framework integrating echocardiography, lung ultrasound, and diaphragm ultrasound may support identification and targeted treatment of the dominant mechanism of weaning failure before extubation. Prospective multicentre validation of the integrated protocol as a whole remains a priority research need.

## 1. Introduction

Weaning from mechanical ventilation is among the most consequential decisions in intensive care medicine. While 80–85% of patients achieve successful extubation at the first attempt, 10–20% fail initial SBTs, and a similar proportion of those successfully extubated require reintubation within 48 h [1,2]. Reintubation is independently associated with increased ICU mortality, nosocomial pneumonia, and prolonged length of stay—outcomes that emphasise the importance of identifying reversible obstacles before extubation is attempted [2,3].

Among the recognised causes of weaning failure, cardiovascular dysfunction is both prevalent and clinically underappreciated. In a seminal prospective cohort, Moschietto et al. demonstrated that deterioration of LV relaxation during an SBT—quantified by tissue Doppler imaging—was the strongest independent predictor of weaning failure [4]. Population-level data from the European multicentre EPWORTH registry of 2729 ventilated patients confirmed that 31% experienced difficult and 14% prolonged weaning, populations in whom cardiac factors are disproportionately represented [3]. The underlying mechanism reflects a fundamental haemodynamic phenomenon: positive pressure ventilation actively unloads the left ventricle, and this protective effect is lost abruptly at extubation. In patients with pre-existing diastolic dysfunction, systolic impairment, or significant mitral regurgitation, the resulting afterload surge may precipitate acute cardiogenic pulmonary oedema—termed weaning-induced pulmonary oedema (WiPO)—within 30 to 90 min [5,6,7,8].

Echocardiography is the only non-invasive modality providing simultaneous assessment of LV and RV systolic function, diastolic filling pressures, valvular pathology, and fluid status at the bedside [7,9,10,11]. Its serial application before and after therapeutic interventions enables both diagnosis and monitoring of therapeutic response—a capability that is essential in a domain where haemodynamic trajectories evolve over hours.

Beyond cardiac assessment, weaning failure is frequently multifactorial. Residual interstitial pulmonary oedema and diaphragm dysfunction—the latter affecting 25–50% of patients after several days of controlled ventilation, with prevalence rates approaching 60% in patients admitted with sepsis—may compound cardiac vulnerability or act independently [12,13,14]. An integrated ultrasound approach that combines echocardiography with lung and diaphragm ultrasound may therefore resolve diagnostic uncertainty in most difficult weaning cases and directly guide the selection and sequencing of targeted therapies.

This narrative review synthesises current evidence on the echocardiographic and integrated ultrasound assessment of weaning failure. It is intended as an educational and clinical reference for intensivists; its recommendations reflect the authors’ synthesis of available evidence and should not be interpreted as formal practice guidelines. Section 4, Section 5 and Section 6 address the underlying physiology and parameter-specific cardiac assessment. Section 7 and Section 8 describe the integrated diagnostic framework and its therapeutic consequences. Section 9 discusses limitations and future perspectives.

## 2. Methods

This article presents a narrative review; it does not follow systematic review methodology, and no formal protocol was registered. The recommendations presented should be understood as a synthesis of the available evidence rather than as formal clinical guidelines. A structured literature search was conducted in PubMed/MEDLINE and EMBASE for publications from January 2000 to April 2026. Pivotal mechanistic studies predating this period were retained for physiological context and were identified through hand-searching of reference lists. Search term combinations included:

1. “Echocardiography” AND (“Weaning” OR “Mechanical Ventilation” OR “Spontaneous Breathing Trial” OR “Extubation”);
2. “Diastolic Dysfunction” AND (“Weaning Failure” OR “Extubation Failure” OR “Left Atrial Pressure”);
3. “Right Ventricular Dysfunction” AND (“Mechanical Ventilation” OR “ARDS” OR “PEEP”);
4. “Lung Ultrasound” AND (“Weaning” OR “Extubation Failure” OR “ICU”);
5. “Diaphragm Ultrasound” AND (“Mechanical Ventilation” OR “Critical Illness” OR “Weaning”).

Eligible study types included prospective and retrospective original research, systematic reviews and meta-analyses, multicentre cohort studies, clinical guidelines, and consensus statements from major critical care and cardiology societies. Case reports and small uncontrolled series without broadly generalisable findings were excluded. Priority was assigned to studies published after 2010 that used validated echocardiographic methodology and reported patient-relevant outcomes such as weaning success, extubation failure, or reintubation rate. A PRISMA-NR flow diagram is provided in Supplementary File S1. A total of 47 publications form the direct evidence base of this review, and 17 key primary studies are summarised in Table 1.

**Table 1:** Characteristics of key included primary studies.

First Author (Year)	Design/n	Population	Primary Parameter	Main Finding/Clinical Relevance
Moschietto et al. (2012) [4]	Prospective cohort n = 41	Mixed ICU; MV > 48 h	E/A ratio, e’ (TDI) during SBT	Deterioration of e’ during SBT was the strongest predictor of weaning failure (AUC 0.88)
Lamia et al. (2009) [5]	Prospective cohort n = 23	ICU; difficult weaning	E/e’, TDI during SBT	E/e’ > 8.5: sensitivity 82%, specificity 91% for elevated PAOP
Caille et al. (2010) [6]	Prospective cohort n = 22	Difficult weaning	Echo LAP profile + PEEP test	Cardiac cause identified in 59%; all cardiac failures had LAP elevation
de Meirelles Almeida et al. (2016) [15]	Systematic review + meta-analysis 10 studies	Mechanically ventilated patients	E/e’, E/A ratio at SBT	Higher E/e’ ratio significantly associated with weaning failure (mean diff +2.65; 95% CI 0.52–4.79)
Bedet et al. (2019) [8]	Prospective multicentre n = 208	First-SBT failures	Echo + BNP + protein	WiPO in 59.6% (liberal definition); WiCI in 20.3%; cardiac failure dominant mechanism
Goudelin et al. (2020) [16]	Prospective cohort n = 59	COPD and/or HFrEF (EF ≤ 40%)	Echo before and during SBT; fluid balance	LV overloading identified by CCE is a key mechanism in WIPO; echo-guided therapy enabled successful extubation in all WIPO patients
Thille et al. (2019) [2]	Multicentre RCT n = 641	High-risk extubation patients	HFNO+NIV vs. HFNO post-extubation	HFNO+NIV reduced reintubation rate at day 7 (11.8% vs. 18.2%, *p* = 0.02)—supports prophylactic NIV in high-risk patients
Béduneau et al. (EPWORTH 2017) [3]	Prospective multicentre n = 2729	General ICU; MV	Clinical weaning classification	55% simple, 31% difficult, 14% prolonged weaning; largest epidemiological dataset
Ferré et al. (2019) [17]	Prospective cohort n = 42/62 SBTs	Planned extubation	ΔB-lines during SBT	ΔB-lines ≥ 6: sensitivity 88%, specificity 88% for WiPO (AUC 0.91)
Bouhemad et al. (2020) [18]	Prospective observational n = 40	Elderly high-risk cardiac patients	Combined cardiac + lung US during SBT	Anterolateral LUS score predicted weaning/extubation failure (AUC 0.79–0.81); combined approach superior to filling pressures alone
Vignon et al. (2023) [7]	Narrative review —	Mixed ICU	Heart–lung interactions, echo-Doppler	Comprehensive review of cardiopulmonary interactions during weaning; LV overload central in WIPO
Goligher et al. (2015) [19]	Prospective cohort n = 191	ICU; MV	Diaphragm TF (M-mode)	TF feasible in 96%; excellent reproducibility (ICC 0.93)
Demoule et al. (2013) [13]	Prospective cohort n = 85	ICU on admission	Magnetic phrenic stimulation	Diaphragm dysfunction in 64% at ICU admission; associated with weaning failure and ICU mortality
Dres et al. (2017) [12]	Prospective cohort n = 85	ICU; MV ≥ 48 h	TF + phrenic stimulation	VIDD in 47%; TF < 29% identified weakness (AUC 0.82)
Goligher et al. (2018) [14]	Prospective cohort n = 211	ICU; MV	Diaphragm atrophy index	Atrophy associated with ICU mortality and prolonged ventilation
Parada-Gereda et al. (2023) [20]	Systematic review + meta-analysis 19 studies; n = 1204	Mechanically ventilated patients	Diaphragm TF, excursion at SBT	TF: sensitivity 85%, specificity 75% for successful weaning (AUC 0.87); excursion AUC 0.87
Mekontso Dessap et al. (2016) [21]	Prospective multicentre n = 752	ARDS; MV	Echo cor pulmonale assessment	ACP in 22.2%; prone positioning significantly reduced ACP prevalence

ACP = acute cor pulmonale; AUC = area under the ROC curve; BNP = brain natriuretic peptide; ICC = intraclass correlation coefficient; LAP = left atrial pressure; MV = mechanical ventilation; PAOP = pulmonary artery occlusion pressure; SBT = spontaneous breathing trial; TDI = tissue Doppler imaging; TF = thickening fraction; VIDD = ventilator-induced diaphragm dysfunction; WiCI = weaning-induced cardiac ischaemia; WiPO = weaning-induced pulmonary oedema.

## 3. Summary of Key Included Primary Studies

Table 1 provides an overview of the 17 key primary observational and interventional studies that directly inform the narrative synthesis of this review. Together, these studies enrolled more than 5000 patients across diverse ICU populations—including medical, surgical, cardiac, and ARDS cohorts—with a median sample size of 84 patients per study (range: 22–2729). The full literature identification and selection process is documented in the PRISMA-NR flow diagram (Supplementary File S1).

## 4. Heart–Lung Interactions During Mechanical Ventilation and Weaning

### 4.1. Effects of Positive Pressure Ventilation on the Right and Left Ventricle

Positive pressure ventilation alters intrathoracic pressure in ways that affect both ventricles simultaneously, yet through distinct and partially opposing mechanisms [22,23]. Recognising these interactions is the physiological prerequisite for echocardiographically guided weaning management.

**Figure 1:**
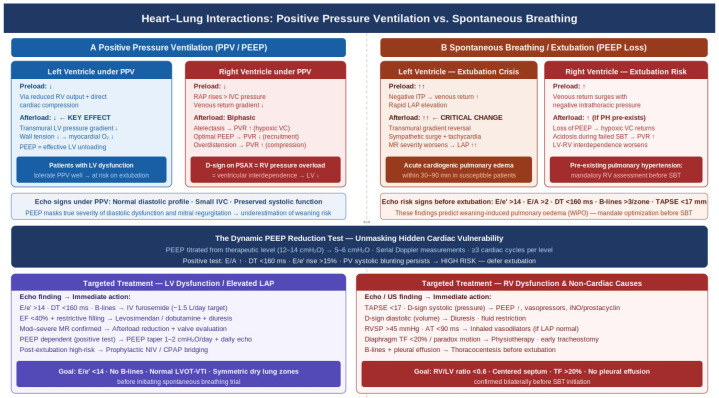
Heart–lung interactions under positive pressure ventilation (PPV, **left panel**)) and at extubation (**right panel**). PEEP unloads the left ventricle by reducing transmural LV pressure. Extubation reverses this effect abruptly, generating an afterload surge and LAP elevation in susceptible patients. Lower panels summarise targeted treatment responses for each haemodynamic phenotype. LV = left ventricle; RV = right ventricle; LAP = left atrial pressure; IAS = interatrial septum; MR = mitral regurgitation; TF = diaphragm thickening fraction; iNO = inhaled nitric oxide.

For the right ventricle, the inspiratory increase in mean airway pressure impedes venous return by raising right atrial pressure relative to the inferior vena cava, thereby reducing RV preload. The effect on RV afterload is biphasic and depends on the state of the lung parenchyma: PEEP applied to atelectatic lungs recruits alveoli, corrects hypoxic pulmonary vasoconstriction, and reduces pulmonary vascular resistance; PEEP applied to already-inflated lungs produces alveolar overdistension, compresses intra-alveolar capillaries, and increases RV afterload [24]. This biphasic relationship explains why PEEP titration in ARDS patients requires sequential echocardiographic surveillance of RV size and septal geometry [25]. (see Figure 1)

For the left ventricle, the clinically decisive effect of PEEP is afterload reduction. LV wall tension is determined by the transmural systolic pressure gradient—the difference between LV intracavitary pressure and pleural pressure. Since PEEP increases pleural pressure proportionally more than it raises LV systolic pressure, the transmural gradient falls, myocardial wall tension decreases, and myocardial oxygen demand is reduced. This mechanism explains why patients with significant diastolic dysfunction, reduced ejection fraction, or moderate-to-severe mitral regurgitation may tolerate positive pressure ventilation remarkably well despite haemodynamically significant cardiac pathology [6,15]. (see Figure 1)

### 4.2. The Transition to Spontaneous Breathing

The haemodynamic changes that occur at extubation are the direct reversal of those described above. Generation of negative pleural pressure during unassisted spontaneous inspiration increases the LV transmural gradient acutely, raising effective afterload. Simultaneously, the augmented venous return that accompanies negative intrathoracic pressure floods a potentially non-compliant left ventricle, elevating end-diastolic filling pressures. These purely mechanical effects are compounded by the metabolic consequences of resumed respiratory work: increased catecholamine release, tachycardia, and rising myocardial oxygen demand, all of which further impair diastolic relaxation and reduce the threshold for decompensation [5,6]. (see Figure 1)

In patients with diastolic dysfunction Grade II or III, or with systolic dysfunction, this convergence of loading changes and metabolic stress may exceed the heart’s compensatory capacity within minutes, generating progressive LAP elevation that manifests clinically as weaning-induced pulmonary oedema (WiPO). The echocardiographic hallmarks—rising E/e’, emergence of IAS bowing, and worsening mitral inflow restrictive pattern—can be identified during the PEEP reduction test before extubation is attempted, enabling preventive rather than reactive management [5,15]. Table 2 summarises the haemodynamic effects of PPV on both ventricles and their echocardiographic correlates.

## 5. Echocardiographic Assessment of Left Atrial Pressure and Diastolic Function

Left atrial pressure is the pivotal echocardiographic target in weaning management. It directly determines the risk of pulmonary oedema upon extubation, guides the intensity and timing of diuretic therapy, and serves as the primary monitoring endpoint during the PEEP reduction test [5,9]. Because no single parameter reliably estimates LAP in isolation—particularly under the confounding influence of mechanical ventilation—a multiparametric approach integrating pulsed Doppler, tissue Doppler, and structural indicators is required [26].

### 5.1. Transmitral Flow: E/A Ratio and Deceleration Time

The transmitral pulsed-wave Doppler profile, recorded at the mitral valve leaflet tips in the apical four-chamber view, is the first component of diastolic assessment. The ratio of the early passive filling wave (E) to the late atrial contraction wave (A) classifies diastolic dysfunction severity: E/A < 1 with deceleration time (DT) > 240 ms indicates impaired LV relaxation with normal LAP (Grade I); E/A 1–2 may represent pseudonormalisation requiring further parameters for disambiguation; E/A > 2 with DT < 160 ms signifies a restrictive filling pattern (Grade III) and reliably identifies elevated LAP regardless of systolic function [26]. In the ICU, tachycardia above 90–100 bpm causes E-A fusion and renders the E/A ratio uninterpretable; under these conditions, E/e’ and pulmonary vein flow become the primary diagnostic tools. (See Table 3)

### 5.2. Tissue Doppler Imaging: The E’ Velocity and E/e’ Ratio

Tissue Doppler imaging (TDI) at the septal and lateral mitral annulus measures early diastolic myocardial relaxation velocity (e’), which is substantially load-independent and reflects intrinsic myocardial compliance. Septal e’ < 7 cm/s and lateral e’ < 10 cm/s indicate impaired relaxation [26]. The E/e’ ratio combines the preload-sensitive E-wave with the preload-independent e’ to estimate LAP: values below 8 suggest normal filling pressures, values above 14 indicate elevated LAP consistent with a pulmonary capillary wedge pressure exceeding 15 mmHg, and the range 8–14 constitutes a diagnostic grey zone that necessitates additional parameters or dynamic testing [26,27].

Two important technical caveats apply in the ICU setting. First, in patients with mitral annular calcification—prevalent in elderly patients—TDI-derived e’ is artefactually reduced, and E/e’ should not be used in isolation. Second, significant mitral regurgitation inflates the E-wave amplitude independently of diastolic function, causing E/e’ to overestimate filling pressure; under these circumstances, greater weight must be placed on structural indices and pulmonary vein flow pattern [26]. (See Table 3)

### 5.3. Pulmonary Vein Flow Pattern and Structural Indices

Under normal conditions, pulmonary vein systolic flow dominates (S > D ratio). Systolic blunting (S < D) indicates elevated left atrial compliance burden and is a reliable secondary marker of elevated LAP, independent of sinus rhythm [26]. Systolic flow reversal is specific for severe mitral regurgitation. The emergence or worsening of systolic blunting during the PEEP reduction test strongly supports a dynamic LAP increase and identifies patients at high risk for WiPO [6].

Structural echocardiographic indices complement the Doppler profile. Rightward bowing of the interatrial septum (IAS) indicates an acute LAP-to-RAP gradient exceeding zero and is a sensitive marker of acutely elevated LAP. Persistent leftward to right bowing on serial assessments implies that LAP consistently exceeds RAP, supporting Grade III diastolic dysfunction. Left atrial dilatation (left atrial volume index, LAVI > 34 mL/m^2^) identifies a chronically elevated LAP burden and is associated with increased weaning failure risk. A distended, non-collapsing inferior vena cava (diameter > 21 mm with < 50% respiratory collapse under mechanical ventilation) reflects systemic venous overload and fluid excess [26]. (See Table 3)

### 5.4. The Dynamic PEEP Reduction Test

Among the available assessment tools, the dynamic PEEP reduction test offers the most direct and clinically informative estimate of cardiac vulnerability to extubation [5,6]. The principle is straightforward: by temporarily removing the afterload-reducing effect of PEEP under controlled monitoring, the test mimics the haemodynamic challenge of extubation before it occurs in an uncontrolled setting. PEEP is reduced from the therapeutic level—typically 12–14 cmH_2_O—to 5–6 cmH_2_O without disconnecting the patient from the ventilator, while serial Doppler measurements are obtained across a minimum of three consecutive cardiac cycles at each PEEP level.

A positive result is defined by any of the following: appearance or worsening of a restrictive E/A pattern, DT falling below 160 ms, E/e’ increasing by more than 15% from baseline, or persistent or worsening systolic blunting on pulmonary vein Doppler. A positive test indicates active PEEP-dependent LV unloading and identifies the patient as high risk for WiPO at extubation. In this setting, a stepwise PEEP reduction strategy—decreasing by 1–2 cmH_2_O per day under daily echocardiographic monitoring—combined with targeted diuresis provides a safer and more controlled pathway to extubation than abrupt wean attempts [6,15]. Transoesophageal echocardiography (TEE) is preferred for this test when available, as it enables continuous uninterrupted Doppler monitoring [28].

## 6. Right Ventricular Function and Pulmonary Circulation

### 6.1. Physiological Vulnerability of the RV During Weaning

The right ventricle is disproportionately vulnerable to the haemodynamic challenges of weaning. Its thin wall (normal 2–5 mm), complex crescent-shaped geometry, and optimisation for low-pressure, high-volume loading render it acutely sensitive to afterload increases that the left ventricle would compensate for without difficulty [25]. The transition from positive to negative intrathoracic pressure at extubation simultaneously increases RV preload through augmented venous return and may elevate RV afterload through the return of hypoxic pulmonary vasoconstriction suppressed by PEEP [22,24]. In patients with pre-existing pulmonary hypertension, right heart failure following ARDS, or RV geometry distorted by chronic lung disease, these concurrent stresses may precipitate acute RV decompensation.

The clinical implications extend beyond isolated RV failure through ventricular interdependence: dilatation of the RV under pressure overload displaces the interventricular septum leftward (D-sign), impairs LV geometry and compliance, and reduces LV preload and stroke volume. The resulting low cardiac output syndrome can closely mimic primary LV failure, and the two require echocardiographic differentiation because their treatments are diametrically opposed [25]. RV dysfunction is present in 20–50% of mechanically ventilated ICU patients and independently predicts weaning failure and mortality when identified on echocardiography [25].

### 6.2. Echocardiographic Assessment of the RV

RV assessment demands a structured multiparametric approach. The apical four-chamber view provides the RV/LV end-diastolic area ratio (normal < 0.6); values approaching or exceeding 1.0 indicate significant RV dilatation and must be confirmed in at least two planes—including the parasternal short-axis and subcostal views—to exclude transducer angulation artefacts that can either overestimate or underestimate RV size [11]. A basal RV diameter exceeding 42 mm and a mid-cavity diameter exceeding 35 mm meet quantitative criteria for dilatation.

Longitudinal systolic function is quantified by TAPSE (Tricuspid Annular Plane Systolic Excursion; normal ≥ 17 mm by M-mode) and by TDI systolic velocity S’ at the lateral tricuspid annulus (normal > 9.5 cm/s). Fractional area change (FAC ≥ 35%) captures both longitudinal and radial contractile components in the apical four-chamber view and is less preload-sensitive than TAPSE. RV free wall strain by speckle tracking (−20% to −28%) provides the highest sensitivity for subclinical RV dysfunction but requires adequate acoustic windows that are not always available in ventilated patients [11,25].

Septal geometry in the parasternal short-axis view allows differentiation of RV loading type, which is essential for treatment selection. Systolic flattening of the interventricular septum (systolic D-sign) indicates RV pressure overload and calls for PEEP optimisation, vasopressor support, and pulmonary vasodilation when LAP is normal. Diastolic septal flattening indicates RV volume overload and is treated with diuresis and fluid restriction. The co-occurrence of systolic and diastolic D-sign components at different phases of the respiratory cycle points to combined pressure-volume overload requiring sequenced, echo-guided titration [24,25]. (See Table 4)

### 6.3. Pulmonary Haemodynamics and Weaning Relevance

Estimation of pulmonary artery systolic pressure (PASP) from the tricuspid regurgitation velocity (RVSP ≈ PASP = 4 × TRV^2^ + estimated RAP) provides a direct measure of RV systolic afterload. RVSP exceeding 45 mmHg warrants further characterisation and targeted management. Pulmonary valve acceleration time (PVAT) below 90 ms and mid-systolic notching of the pulmonary flow profile (flying-W sign) are specific markers of elevated pulmonary vascular resistance and distinguish pre-capillary from post-capillary pulmonary hypertension—a distinction of direct therapeutic significance [25]. Inhaled nitric oxide and inhaled prostacyclin are appropriate for isolated RV pressure overload with normal LAP but would exacerbate cardiogenic pulmonary oedema if applied in the context of elevated filling pressures; echocardiography is the only practical tool for making this distinction at the bedside.

## 7. Integrated Ultrasound Approach

### 7.1. Transthoracic Versus Transesophageal Echocardiography

For most weaning-relevant assessments, transthoracic echocardiography (TTE) provides adequate diagnostic quality and represents the practical standard. LV and RV systolic function, diastolic profile, RV loading state, IVC, and structural indices are obtainable in the large majority of patients, and TTE can be performed and repeated at any time without patient preparation or procedural risk [11,28]. Transoesophageal echocardiography (TEE) is indicated when TTE acoustic windows are insufficient—as in obese patients, those with thoracic dressings or drains, and patients with severe COPD—or when precise quantification of valvular regurgitation is needed for therapeutic planning (MitraClip candidacy, valve surgery). For the dynamic PEEP reduction test, TEE provides uninterrupted, continuous Doppler monitoring that is technically superior to serial TTE measurements [5,28]. In ARDS patients with refractory hypoxaemia and suspected acute cor pulmonale or intracardiac shunt, TEE is the recommended modality [29].

### 7.2. Lung Ultrasound: B-Lines and Pleural Pathology

Lung ultrasound B-lines—hyperechoic, laser-like artefacts arising from the fluid-thickened interlobular septa—provide a semi-quantitative, real-time measure of extravascular lung water that directly complements echocardiographic LAP estimation [30]. More than three B-lines per zone in bilateral distribution constitutes pathological interstitial oedema and predicts postextubation respiratory failure, particularly when integrated with cardiac ultrasound assessment [17,18]. Ferré et al. demonstrated that B-line quantification during a successful SBT identifies patients at risk for subsequent WiPO with an area under the receiver operating characteristic curve of 0.92, superior to any clinical variable alone [17]. B-lines respond to diuresis within hours, making them an ideal near-real-time treatment monitoring tool during pre-extubation optimisation.

The BLUE (Bedside Lung Ultrasound in Emergency) protocol applies a structured scanning approach across six thoracic zones to distinguish the mechanism of acute respiratory failure: bilateral B-lines indicate cardiogenic oedema and mandate echocardiographic assessment of LAP; unilateral B-lines or consolidation with air bronchograms suggest pneumonia as a weaning obstacle; anterior A-lines with bilateral absence of lung sliding point to pneumothorax and require immediate clinical correlation [30]. Pleural effusions, identified as echo-free collections posterior to the descending aorta on the parasternal long-axis view, produce compressive atelectasis, impair diaphragm excursion, and reduce functional residual capacity. Thoracocentesis before extubation reduces the work of breathing and improves weaning success rates when effusion volume exceeds approximately 200 mL per side.

### 7.3. Diaphragm Ultrasound: Thickening Fraction and Excursion

Diaphragm dysfunction is an independent, non-cardiac cause of weaning failure that is frequently missed without dedicated ultrasound assessment. It encompasses two overlapping but distinct entities: pre-existing diaphragm dysfunction present at ICU admission and ventilator-induced diaphragm dysfunction (VIDD) acquired during mechanical ventilation. Demoule et al. demonstrated by magnetic phrenic nerve stimulation that diaphragm dysfunction is present at ICU admission in 64% of patients, particularly those with sepsis, and is associated with prolonged ventilation and increased mortality [13]. VIDD itself results from a combination of disuse atrophy under controlled ventilation, oxidative stress at the myofibril level, and sedation-related suppression of respiratory muscle activity. Histological studies have documented type I and II fibre atrophy within 18–24 h of controlled ventilation in human diaphragm biopsies. Clinically relevant VIDD—defined by TF below 29%—affects 25–50% of patients ventilated beyond 48 h and is detectable before clinical symptoms emerge [12,14,19]. The thickening fraction (TF) is measured with a high-frequency linear probe at the zone of apposition along the anterior axillary line using M-mode: TF = (end-inspiratory thickness − end-expiratory thickness)/end-expiratory thickness × 100%. A TF below 20% indicates diaphragm weakness; a TF of 0% or paradoxical thinning during inspiration signifies complete paresis [19]. Complementarily, a low-frequency phased-array probe positioned subcostally measures M-mode excursion: normal tidal excursion is 1.5–2.5 cm and deep inspiratory excursion is 8–14 cm.

Bilateral measurement is mandatory. Unilateral phrenic palsy—a recognised complication of cardiac and thoracic surgery, mediastinal instrumentation, or cervical procedures—will be missed without side-by-side comparison and is commonly misinterpreted as patient non-cooperation during breathing trials. In a prospective cohort, VIDD identified by serial TF measurement was independently associated with longer mechanical ventilation duration and higher ICU mortality [14]. When bilateral VIDD is identified, the differential diagnosis must include critical illness polyneuropathy and myopathy, electrolyte depletion (phosphate, magnesium, potassium), malnutrition, and primary neuromuscular disease. Targeted physiotherapy, optimisation of metabolic prerequisites, and early tracheostomy in cases where rapid recovery is unlikely represent evidence-informed management strategies [12,14].

## 8. Echocardiography-Guided Weaning Strategy

### 8.1. Pre-SBT Risk Stratification and Assessment

Risk stratification should precede formal echocardiographic evaluation and determine the urgency and depth of the ultrasound workup. High-risk patients—those with documented diastolic dysfunction Grade ≥ II, ejection fraction < 40%, moderate-to-severe mitral or aortic regurgitation, pulmonary hypertension, or clinical evidence of fluid overload—require a complete pre-SBT assessment comprising the full echocardiographic LAP profile, multiparametric RV evaluation, lung ultrasound, and bilateral diaphragm assessment before any trial of spontaneous breathing is initiated.

**Figure 2:**
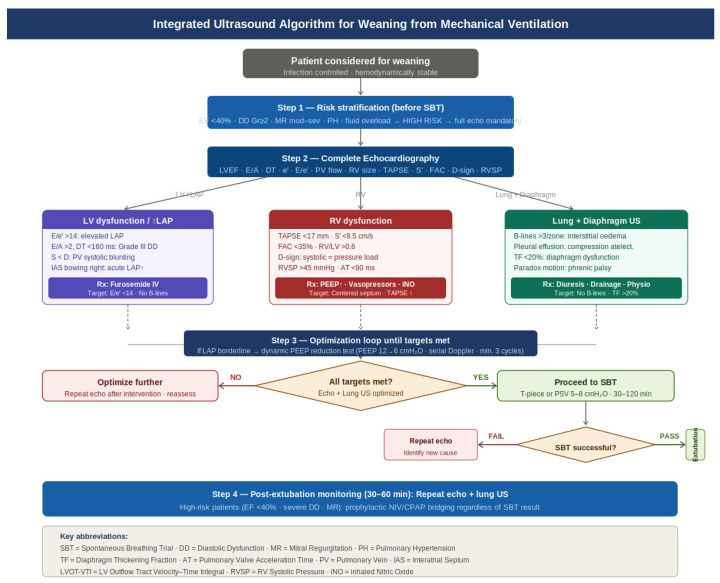
Integrated ultrasound algorithm for weaning from mechanical ventilation. The four-step sequential protocol proceeds from risk stratification through complete echocardiographic and ultrasound assessment, optimisation of identified targets, and post-extubation monitoring. High-risk patients receive prophylactic NIV/CPAP after extubation regardless of SBT outcome. This algorithm is presented as a conceptual decision-support framework derived from current evidence and pathophysiological reasoning; it has not yet undergone prospective multicentre validation. The dynamic PEEP reduction test (Step 3) likewise remains an exploratory assessment that is not yet standardised across centres. SBT = spontaneous breathing trial; LAP = left atrial pressure; NIV = non-invasive ventilation; CPAP = continuous positive airway pressure; iNO = inhaled nitric oxide; TF = diaphragm thickening fraction.

If B-lines exceed three per zone bilaterally, targeted diuresis should be initiated and lung ultrasound repeated after 12–24 h before attempting an SBT. When LAP parameters fall within the grey zone (E/e’ 8–14) and clinical uncertainty remains, the dynamic PEEP reduction test should be performed. A positive test mandates continued optimisation; a negative result with stable or improving diastolic parameters permits SBT initiation. (See Figure 2)

### 8.2. During and After the SBT

Clinical deterioration during an SBT—defined as tachypnoea > 35/min, tachycardia, new-onset hypertension, diaphoresis, or SpO_2_ decline—should prompt immediate echocardiographic assessment to characterise the mechanism. The reappearance of B-lines, acute LAP elevation on Doppler, or new RV loading changes identifies cardiac weaning failure and mandates return to mechanical ventilation with targeted intervention before the next attempt.

After successful extubation, a follow-up echo and lung ultrasound 30–60 min later detects delayed WiPO, which may emerge after the clinical tolerance window of the SBT has closed. In carefully selected high-risk patients—those with EF < 40%, severe diastolic dysfunction, or PEEP-dependence identified on pre-extubation testing—prophylactic non-invasive ventilation (NIV) or continuous positive airway pressure (CPAP) may be considered as a post-extubation bridge. The supporting evidence derives primarily from a single multicentre randomised controlled trial restricted to patients older than 65 years or with underlying cardiac or respiratory disease [2]; broader generalisability remains to be established, and the strategy should be individualised rather than applied universally. Serial lactate and NT-proBNP trajectories complement the ultrasound assessment during the post-extubation monitoring period. (See Figure 2)

## 9. Clinical Implications and Therapeutic Consequences

The clinical value of pre-extubation echocardiography extends beyond diagnosis: it generates specific, measurable therapeutic targets whose achievement before SBT directly predicts extubation success. The therapeutic response to directed interventions can be monitored non-invasively using the same echocardiographic parameters that identified the problem, making the assessment inherently iterative and self-confirming.

### 9.1. Elevated LAP and Diastolic Dysfunction

When elevated LAP is identified pre-extubation, intravenous diuresis targeting a net negative fluid balance of 1.0–1.5 L per day is the primary intervention. The echocardiographic treatment targets are E/e’ below 14, disappearance of IAS bowing, and lung ultrasound clearance of bilateral B-lines. These endpoints can be reassessed within hours of starting diuresis, enabling same-shift decision-making. Furosemide is the standard first-line agent; in patients with renal impairment or diuretic resistance, combination with acetazolamide or torsemide may be required. Excessive diuresis in patients who are simultaneously vasopressor-dependent can precipitate haemodynamic deterioration and must be guided by IVC and LVOT-VTI monitoring.

In patients where systolic dysfunction coexists with diastolic impairment (EF < 40%, restrictive filling pattern), inotropic support with levosimendan or dobutamine in combination with diuresis may be required. The echocardiographic monitoring target in this scenario is an improvement in LVOT-VTI alongside normalisation of the LAP profile [15]. A positive PEEP reduction test in the context of EF < 40% mandates a stepwise PEEP reduction strategy—typically decreasing PEEP by 1–2 cmH_2_O every 24 h under daily echocardiographic monitoring—rather than an abrupt SBT.

### 9.2. RV Dysfunction and Pulmonary Hypertension

Management of RV dysfunction during weaning requires differentiation of loading type, as established by the septal geometry assessment described in Section 5. Pressure overload (systolic D-sign) is addressed by PEEP optimisation to correct hypoxic pulmonary vasoconstriction, vasopressor support to maintain systemic MAP above mean pulmonary artery pressure (thus preserving RV coronary perfusion), and inhaled vasodilators (nitric oxide, prostacyclin) when LAP is confirmed to be normal [24,25]. Volume overload (diastolic D-sign) is managed by diuresis and fluid restriction, targeting a reduction in the RV/LV area ratio below 0.6 and centring of the interventricular septum. Veno-arterial ECMO represents the treatment of last resort for refractory RV failure in patients in whom myocardial recovery is anticipated.

### 9.3. Fluid Overload and Non-Cardiac Obstacles

Fluid accumulation is a common and modifiable weaning obstacle that extends beyond its cardiac consequences. Post-resuscitation fluid overload generates pleural effusions that restrict diaphragm excursion, increases abdominal pressure that limits tidal breathing, and sustains pulmonary interstitial oedema that impairs gas exchange. When B-lines persist despite echocardiographic evidence of normal LAP, the extravascular lung water may be non-cardiogenic in origin (ARDS, inflammatory) and should not be treated with additional diuresis. The combination of B-line quantification and echocardiographic LAP assessment distinguishes cardiogenic from non-cardiogenic oedema in the majority of cases.

Diaphragm dysfunction identified by TF below 20% or paradoxical motion should prompt optimisation of metabolic prerequisites (correction of phosphate, magnesium, and potassium deficiencies), reduction in sedation to the minimum required level, and targeted respiratory physiotherapy. In patients with documented phrenic palsy or severe bilateral VIDD where rapid recovery is unlikely, early tracheostomy is preferable to prolonged translaryngeal intubation, allowing ventilatory support to be continued with lower sedation requirements and enabling progressive spontaneous breathing trials [12,14].

### 9.4. Weaning in Patients with Significant Mitral Regurgitation: A Clinical Scenario Requiring Special Attention

Patients with moderate-to-severe mitral regurgitation (MR) represent a particularly vulnerable subgroup during weaning, and their cardiac risk is systematically underestimated under mechanical ventilation for two interconnected reasons. First, PEEP reduces LV afterload, thereby lowering the transvalvular pressure gradient across the mitral valve and effectively suppressing the regurgitant volume. Colour Doppler at high PEEP (e.g., 12–14 cmH_2_O) may therefore reveal only mild-to-moderate MR, substantially underestimating the true regurgitant severity that emerges once ventilatory support is removed. Second, the haemodynamic consequences of MR at extubation compound rapidly: as negative pleural pressure increases LV afterload, the effective regurgitant orifice area expands, forward stroke volume falls, and LAP rises through both the mechanical surge and the increased regurgitant fraction. The concurrent catecholamine surge—driven by resumed respiratory work and potential dyspnoea—further worsens MR through tachycardia-induced diastolic shortening and adrenergic annular dilation.

*Clinical vignette:* A 61-year-old man with Non-STEMI-related ventricular dysfunction (EF ~ 35%), moderate-to-severe MR, and moderate aortic stenosis underwent attempted extubation after stabilisation on PEEP 14 cmH_2_O. TEE at high PEEP demonstrated what appeared to be moderate MR with a single jet into the left atrium; E/A was 2.3 with DT 220 ms and S/D blunting. On PEEP reduction to 6 cmH_2_O, E/A rose to 2.6 and DT fell to 164 ms—confirming dynamic LAP instability. Extubation was deferred. A stepwise PEEP reduction strategy (1–2 cmH_2_O per day) under daily TEE monitoring, combined with furosemide-guided diuresis and heart rate reduction, achieved successful extubation after 6 days.

Key echocardiographic principles apply to MR patients planning weaning. TEE-based MR quantification at a standardised intermediate PEEP level (8–10 cmH_2_O)—using vena contracta width and PISA-derived effective regurgitant orifice area—provides the most accurate assessment of true MR severity. A vena contracta > 7 mm or EROA > 0.4 cm^2^ at this intermediate PEEP suggests clinically significant MR that is likely to worsen at extubation. In selected patients with severe MR causing refractory WiPO despite optimal medical management, valve-directed intervention (MitraClip transcatheter mitral repair or surgical mitral valve surgery) may represent a prerequisite for successful weaning, consistent with current European and American valvular disease guidelines [26]. (See Table 5)

## 10. Limitations and Future Perspectives

Several methodological and technical limitations must be appreciated when applying echocardiographic assessment during weaning in clinical practice.

Validation gap in the critically ill: The ASE/EACVI 2016 diastolic function guidelines were derived from and validated in ambulatory, haemodynamically stable outpatient cohorts [26]. Their performance in mechanically ventilated patients is different: PEEP modifies all transmitral and TDI-derived parameters independently of underlying cardiac function, and absolute thresholds cannot be applied without accounting for the ventilatory state. The evidence for E/e’, DT, and E/A as predictors of weaning failure derives primarily from single-centre prospective cohort studies with limited sample sizes; multicentre validation is needed before these thresholds can be regarded as universally applicable [26].

Parameter-specific confounders: Tachycardia above 90–100 bpm causes E-A fusion, rendering the transmitral ratio uninterpretable and necessitating reliance on E/e’ alone. Mitral annular calcification, prevalent in elderly patients, invalidates TDI-derived e’ measurements. Significant mitral regurgitation amplifies the E-wave independently of filling pressure, making E/e’ unreliable for LAP estimation; in this situation, greater reliance on pulmonary vein flow and dynamic testing is required. Atrial fibrillation eliminates the A-wave and requires DT-based and E/e’-based assessment without E/A [26].

Load dependency and dynamic change: All Doppler-derived cardiac parameters are influenced by loading conditions at the time of measurement. Static assessments performed at high PEEP systematically underestimate the severity of diastolic dysfunction by masking the haemodynamic instability that will emerge at extubation. This load dependency is, paradoxically, the basis for the diagnostic value of the PEEP reduction test but also explains why single time-point measurements—without dynamic provocation or serial comparison—have limited predictive accuracy.

Image quality and operator variability: Adequate acoustic windows are not available in all ventilated patients; obesity, subcutaneous emphysema, thoracic wounds, and mechanical ventilation itself reduce TTE image quality. Inter-observer variability in visual RV/LV ratio assessment and manual TAPSE measurement underscores the need for structured acquisition protocols and quantitative measurements where possible. Diaphragm ultrasound TF measurement carries its own reproducibility challenges when the diaphragm is thin or when respiratory movement is minimal under controlled ventilation.

Review methodology: This article is intentionally structured as a narrative rather than a systematic review. The available evidence base is methodologically heterogeneous—comprising prospective single-centre cohorts, retrospective multicentre studies, expert consensus statements, and society guidelines—and is not amenable to meta-analytic pooling without substantial clinical and statistical heterogeneity. Each component of the integrated protocol has been studied independently across different patient populations and ventilatory contexts, precluding a unified PICO-based synthesis. Formal study quality appraisal was not performed, publication bias in favour of positive echocardiographic associations cannot be excluded, and no prospective multicentre trial has yet validated the complete integrated ultrasound protocol described here as a whole. The evidence base is assembled from individual studies of component assessments performed in heterogeneous patient populations and ventilatory contexts.

### Future Perspectives

Several directions represent priorities for future research and clinical implementation. Prospective multicentre trials evaluating the impact of integrated echo-guided weaning strategies on reintubation rates, ICU length of stay, and mortality are needed; the current evidence is derived from component assessments performed in heterogeneous populations, and no trial has yet evaluated the complete integrated protocol prospectively. Artificial intelligence-assisted diastolic function grading using deep learning models trained on transmitral Doppler and TDI datasets, together with automated RV volumetry using real-time three-dimensional echocardiography, may reduce operator dependency and improve reproducibility in ICU environments where trained echocardiographers are not continuously available. Consensus on procedural parameters for the dynamic PEEP reduction test—target PEEP level, duration, specific Doppler endpoints, and safety stopping criteria—is needed to enable cross-study comparison and formal validation. Emerging wearable patch-based ultrasound technology capable of continuous B-line monitoring may enable real-time post-extubation surveillance, addressing a critical monitoring gap in the first hour after extubation. Finally, the role of diaphragm pacing, neuromuscular electrical stimulation, and pressure-supported ventilation titrated by diaphragm ultrasound TF feedback to prevent VIDD warrants prospective evaluation, and emerging data on diaphragm electrical activity monitoring (EAdi via oesophageal catheter) as a complement to ultrasound may further refine weaning readiness assessment.

## 11. Conclusions

Weaning failure is a cardinal clinical challenge in the ICU, and cardiovascular dysfunction—primarily diastolic dysfunction with elevated LAP—is its most prevalent and most underappreciated mechanism. The vulnerability of cardiac patients to extubation is masked during positive pressure ventilation by the active LV afterload reduction that PEEP provides; extubation removes this protection abruptly and predictably in those whose myocardial compliance is insufficient to accommodate the resulting haemodynamic stress.

Echocardiography is the only bedside tool capable of characterising this vulnerability before clinical decompensation occurs. The multiparametric LAP assessment—integrating E/e’, DT, e’, pulmonary vein flow, and structural indices—provides pre-extubation phenotyping that directly guides the timing and intensity of diuresis. The dynamic PEEP reduction test unmasks latent LAP instability and identifies patients who require a gradual, echo-monitored PEEP-weaning strategy rather than an abrupt SBT. RV dysfunction, identified through TAPSE, D-sign, and RVSP, requires separate assessment because its treatment is fundamentally different from that of LV failure and because ventricular interdependence means that unrecognised RV overload produces a clinical picture mimicking primary LV dysfunction.

The integration of lung ultrasound B-line quantification and bilateral diaphragm TF measurement into the echocardiographic assessment captures the non-cardiac contributors to weaning failure—residual interstitial oedema and respiratory muscle dysfunction—within a single bedside protocol. Each modality generates quantitative, measurable targets whose achievement before extubation can be monitored within hours of initiating therapy, enabling iterative, evidence-based optimisation rather than fixed protocol application.

Future research should focus on the prospective multicentre validation of the integrated protocol as a whole, the development of standardised training and competency frameworks for its application, and the investigation of novel echocardiographic indices—including real-time three-dimensional RV volumetry and artificial intelligence–assisted diastolic grading—that may improve reliability and reduce operator dependency in the ICU environment.

## Figures and Tables

**Table 2:** Haemodynamic effects of positive pressure ventilation on both ventricles and echocardiographic correlates.

Parameter	RV Under PPV	LV Under PPV	Weaning Risk on PEEP Loss
Preload	↓ Venous return ↓ (RAP > IVC pressure)	↓ Via reduced RV output + direct compression	RV: Preload surge with negative ITP
Afterload	Biphasic: ↓ if recruitment, ↑ if overdistension	↓ Transmural LV gradient ↓ → wall tension ↓	LV: Afterload surge → LAP elevation
Echo sign	D-sign (PSAX) if overloaded; IVC plethora	Masked diastolic dysfunction; falsely normal E/e’	Normal echo during PPV ≠ safe to extubate
Key message	PEEP titration needs RV monitoring	PEEP unloads LV: this benefit is lost at extubation	Pre-extubation assessment mandatory in all high-risk patients

RV = right ventricle; LV = left ventricle; RAP = right atrial pressure; IVC = inferior vena cava; ITP = intrathoracic pressure; LAP = left atrial pressure; PSAX = parasternal short-axis view; PEEP = positive end-expiratory pressure. Arrows: up: increase from norm and down: decrease from norm.

**Table 3:** Echocardiographic LAP profile: interpretation and clinical action before spontaneous breathing trial.

Echo Pattern	Interpretation	Clinical Decision
E/A > 2 · DT < 160 ms · S < D · E/e’ > 14 · IAS bowing right	Grade III DD—confirmed elevated LAP	Defer SBT → IV furosemide (target −1.5 L/day)
E/A 1–2 · e’ reduced · S ≈ D · E/e’ 8–14	Probable elevated LAP—grey zone	Dynamic PEEP reduction test · serial monitoring
E/A < 1 · DT > 240 ms · S > D · E/e’ < 8	Normal LAP	Proceed to SBT · echo surveillance during trial
AF · E/A uninterpretable · DT < 140 ms	Elevated LAP probable (no A-wave)	Rate control + furosemide; use DT and E/e’
Significant MR · high E/e’	E-wave inflated by MR volume—E/e’ unreliable	Rely on PV systolic blunting + PEEP test; MR quantification by TEE

SBT = spontaneous breathing trial; DD = diastolic dysfunction; AF = atrial fibrillation; MR = mitral regurgitation; PV = pulmonary vein; IAS = interatrial septum. Arrows: up: increase from norm and down: decrease from norm.

**Table 4:** Echocardiographic parameters for RV function assessment during weaning from mechanical ventilation.

Parameter	Normal	Threshold	Interpretation and Caveats
TAPSE	≥17 mm	<17 mm	Longitudinal RV dysfunction; preload-dependent; may be falsely normal with high preload
S’ (TDI)	>9.5 cm/s	<9.5 cm/s	Less load-dependent; requires adequate TDI window; angle-dependent
FAC	≥35%	<35%	Integrates longitudinal and radial function; planimetric; less preload-sensitive
RV/LV area ratio	<0.6	≥1.0 (RV = LV)	Severe dilatation; confirm in ≥2 planes to avoid axis error
D-sign (PSAX)	Absent	Systolic = pressure Diastolic = volume	Systolic: PEEP↑, vasopressors, iNO if LAP normal Diastolic: diuresis
RVSP (TRV)	<35 mmHg	>45 mmHg	RV pressure overload; rule out PE when clinically suspected
PVAT	>100 ms	<90 ms (notch)	Elevated PVR; flying-W sign indicates pulmonary hypertension
RV free wall strain	−20 to −28%	>−20%	Most sensitive; technically demanding; valuable for serial monitoring

TAPSE = Tricuspid Annular Plane Systolic Excursion; TDI = Tissue Doppler Imaging; FAC = Fractional Area Change; PSAX = parasternal short-axis; RVSP = RV systolic pressure; TRV = tricuspid regurgitation velocity; PVR = pulmonary vascular resistance; PVAT = pulmonary valve acceleration time; PE = pulmonary embolism; iNO = inhaled nitric oxide. Arrows: up: increase from norm and down: decrease from norm.

**Table 5:** Echo and ultrasound phenotypes, therapeutic responses, and monitoring targets.

Phenotype (Echo/US Finding)	Diagnosis	First-Line Intervention	Monitoring Target
E/e’ > 14 · DT < 160 ms · bilateral B-lines	Elevated LAP—cardiogenic oedema	IV furosemide − 1.5 L/day	E/e’ < 14 · B-line clearance before SBT
EF < 40% + restrictive filling + B-lines	Systolic and diastolic dysfunction	Levosimendan or dobutamine + diuresis	EF ↑ · LVOT-VTI ↑ · E/e’ ↓
TAPSE < 17 + D-sign systolic + RVSP > 45	RV pressure overload	PEEP ↑ · vasopressors · iNO if LAP normal	Centred septum · TAPSE ↑ · RVSP ↓
TAPSE < 17 + D-sign diastolic + RV/LV ≥ 1	RV volume overload	Diuresis · fluid restriction	RV/LV < 0.6 · diastolic D-sign resolved
B-lines + large pleural effusion	Fluid overload + compressive atelectasis.	Diuresis + thoracocentesis before SBT	B-line-free zones · no effusion
Diaphragm TF < 20% bilateral	Ventilator-induced diaphragm dysfunction	Electrolyte repletion · physio · early tracheostomy if severe	TF > 20% bilateral · excursion ≥ 1.5 cm
TF = 0% unilateral + paradox motion	Phrenic palsy (e.g., post-surgical)	Early tracheostomy + unilateral physio	Serial TF and excursion monitoring

LAP = left atrial pressure; SBT = spontaneous breathing trial; EF = ejection fraction; LVOT-VTI = LV outflow tract velocity-time integral; TAPSE = Tricuspid Annular Plane Systolic Excursion; RVSP = RV systolic pressure; TF = diaphragm thickening fraction; iNO = inhaled nitric oxide. Arrows: up: increase from norm and down: decrease from norm.

## Data Availability

No new data were generated or analysed in this study. Data sharing is not applicable to this article.

## Supplementary Materials

The following supporting information can be downloaded at: https://www.mdpi.com/article/10.3390/diagnostics16111709/s1, Figure S1: PRISMA-NR Literature Identification and Selection Flow Diagram; Table S1: Characteristics of Key Included Studies.
