# Supplementary material for: Beyond the Spontaneous Breathing Trial: Echocardiographic and Integrated Ultrasound Assessment During Weaning from Mechanical Ventilation
Source: Diagnostics (Basel). 2026 Jun 2;16(11):1709. doi: 10.3390/diagnostics16111709 (PMC13256540; doi:10.3390/diagnostics16111709)
Supplement: Supplementary file 1 [file diagnostics-16-01709-s001.zip › diagnostics-4311745-supplementary.pdf]

SUPPLEMENTARY FILE 1

PRISMA for Narrative Reviews (PRISMA-NR) Flow Diagram and Table S1: Characteristics of Key Included Studies

Manuscript: Beyond the Spontaneous Breathing Trial: Echocardiographic and Integrated Ultrasound Assessment During Weaning from Mechanical Ventilation

Journal: Diagnostics (MDPI)

Authors: Saeed Torabi, Philipp K. Omuro — University Hospital of Cologne

Note: This review is a narrative (not systematic) review. The diagram below is adapted from PRISMA principles to illustrate the literature identification and selection process, consistent with transparent reporting practices for narrative reviews. Formal study quality appraisal and meta-analytic pooling were not performed.

Figure S1. PRISMA-NR Literature Identification and Selection Flow Diagram

PHASE 1 — IDENTIFICATION

PubMed / MEDLINE	EMBASE	Hand search / Guidelines
n = 856	n = 312	n = 47

▼

Total records identified

n = 1,215

▼

After duplicate removal

n = 893

▼

PHASE 2 — TITLE / ABSTRACT SCREENING

Screened (title + abstract)	Excluded (title/abstract): n = 714
n = 893	• Not related to weaning / echo / US<br>• Animal / paediatric studies<br>• Language other than English or German

▼

PHASE 3 — FULL-TEXT ELIGIBILITY

Assessed for full-text eligibility	Excluded (full text): n = 132
n = 179	• Case reports / <10 patients<br>• No weaning outcome reported<br>• Non-validated echo methodology<br>• Duplicate reports / cohort overlap

▼

PHASE 4 — INCLUDED IN NARRATIVE SYNTHESIS

Included in narrative synthesis<br>**n = 47**	**Of which:**<br>• Key included studies (Table S1): 17<br>• Additional primary cohorts: 9<br>• Clinical guidelines / consensus: 8<br>• Narrative reviews / expert opinion: 13

Note: Numbers reflect the structured literature search conducted in PubMed/MEDLINE and EMBASE from January 2000 to April 2026. Foundational physiological studies predating this period (e.g., Michard & Teboul 2000, Pinsky 2007) were identified through hand-searching of reference lists and included in the narrative synthesis for physiological context. This diagram is adapted from PRISMA guidelines for transparent reporting of narrative reviews (PRISMA-NR); it does not imply systematic review methodology, and formal quality appraisal was not performed.

Table S1. Characteristics of Key Included Studies

The following 17 key included observational, interventional, and synthesis-level studies directly inform the narrative synthesis. Study selection prioritised prospective designs with validated echocardiographic or ultrasound methodology, multicentre RCTs and meta-analyses where available, and patient-relevant outcomes (weaning success, extubation failure, reintubation, ICU mortality). This table mirrors Table 1 in the main manuscript and provides the same information in expanded format for reference.

First Author (Year)	Study Design	n	Patient Population	Primary Echo / US Parameter	Main Finding
Moschietto et al. (2012) [4]	Prospective cohort	41	Mixed ICU, MV >48 h	E/A ratio, e' (TDI) during SBT	Deterioration of e' during SBT was the strongest independent predictor of weaning failure (AUC 0.88)
Lamia et al. (2009) [5]	Prospective cohort	23	ICU, difficult weaning	E/e', TDI during SBT	E/e' >8.5: sensitivity 82%, specificity 91% for elevated PAOP
Caille et al. (2010) [6]	Prospective cohort	22	Difficult weaning	Echo LAP profile + PEEP test	Cardiac cause identified in 59%; all cardiac failures had LAP elevation
de Meirelles Almeida et al. (2016) [15]	Systematic review + meta-analysis (10 studies)	n/a	Mechanically ventilated patients	E/e', E/A ratio at SBT	Higher E/e' ratio significantly associated with weaning failure (mean difference +2.65; 95% CI 0.52–4.79)
Bedet et al. (2019) [8]	Prospective multicentre	208	First-SBT failures	Echo + BNP + protein	WiPO in 59.6% (liberal definition); WiCI in 20.3%; cardiac failure dominant mechanism
Goudelin et al. (2020) [16]	Prospective cohort	59	COPD and/or HFrEF (EF ≤40%)	Echo before and during SBT; fluid balance	LV overloading identified by CCE is the key mechanism in WIPO; echo-guided therapy enabled successful extubation in all WIPO patients
Thille et al. (2019) [2]	Multicentre RCT	641	High-risk extubation patients	HFNO+NIV vs HFNO post-extubation	HFNO+NIV reduced reintubation at day 7 (11.8% vs 18.2%, p = 0.02) — supports prophylactic NIV in carefully selected high-risk patients
Béduneau et al. (EPWORTH 2017) [3]	Prospective multicentre	2,729	General ICU, MV	Clinical weaning classification	55% simple, 31% difficult, 14% prolonged weaning; largest epidemiological dataset
Ferré et al. (2019) [17]	Prospective cohort	42 (62 SBTs)	Planned extubation	ΔB-lines during SBT	ΔB-lines ≥6: sensitivity 88%, specificity 88% for WiPO (AUC 0.91)
Bouhemad et al. (2020) [18]	Prospective observational pilot	40	Elderly high-risk cardiac patients	Combined cardiac + lung US during SBT	Anterolateral LUS score predicted weaning/extubation failure (AUC 0.79–0.81); combined approach superior to filling pressures alone
Vignon et al. (2023) [7]	Narrative review	—	Mixed ICU	Heart–lung interactions, echo-Doppler	Comprehensive review of cardiopulmonary interactions during weaning; LV overload central in WIPO

First Author (Year)	Study Design	n	Patient Population	Primary Echo / US Parameter	Main Finding
Goligher et al. (2015) [19]	Prospective cohort	191	ICU, MV	Diaphragm TF (M-mode)	TF feasible in 96%; excellent reproducibility (ICC 0.93)
Demoule et al. (2013) [13]	Prospective cohort	85	ICU on admission	Magnetic phrenic stimulation	Diaphragm dysfunction in 64% at ICU admission; associated with weaning failure and ICU mortality
Dres et al. (2017) [12]	Prospective cohort	85	ICU, MV ≥48 h	TF + phrenic stimulation	VIDD in 47%; TF <29% identified weakness (AUC 0.82)
Goligher et al. (2018) [14]	Prospective cohort	211	ICU, MV	Diaphragm atrophy index	Atrophy associated with ICU mortality and prolonged ventilation
Parada-Gereda et al. (2023) [20]	Systematic review + meta-analysis (19 studies)	1,204	Mechanically ventilated patients	Diaphragm TF, excursion at SBT	TF: sensitivity 85%, specificity 75% for successful weaning (AUC 0.87); excursion AUC 0.87
Mekontso Dessap et al. (2016) [21]	Prospective multicentre	752	ARDS, MV	Echo cor pulmonale assessment	ACP in 22.2%; prone positioning significantly reduced ACP prevalence

Abbreviations: ACP = acute cor pulmonale; ARDS = acute respiratory distress syndrome; AUC = area under the receiver operating characteristic curve; BNP = brain natriuretic peptide; CCE = critical care echocardiography; CI = confidence interval; COPD = chronic obstructive pulmonary disease; DT = deceleration time; EF = ejection fraction; HFNO = high-flow nasal oxygen; HFrEF = heart failure with reduced ejection fraction; ICC = intraclass correlation coefficient; ICU = intensive care unit; LAP = left atrial pressure; LUS = lung ultrasound; LV = left ventricle; MV = mechanical ventilation; NIV = non-invasive ventilation; OR = odds ratio; PAOP = pulmonary artery occlusion pressure; PEEP = positive end-expiratory pressure; RCT = randomised controlled trial; SBT = spontaneous breathing trial; TDI = tissue Doppler imaging; TF = thickening fraction; US = ultrasound; VIDD = ventilator-induced diaphragm dysfunction; WiCI = weaning-induced cardiac ischaemia; WiPO / WIPO = weaning-induced pulmonary oedema.

Cross-reference: Reference numbers in square brackets correspond to the main reference list of the manuscript.
